# Advances in Drug Design of Radiometal-Based Imaging Agents for Bone Disorders

**DOI:** 10.1155/2011/537687

**Published:** 2011-12-15

**Authors:** Kazuma Ogawa, Hideo Saji

**Affiliations:** ^1^Division of Pharmaceutical Sciences, Graduate School of Natural Science and Technology, Kanazawa University, Kakuma-machi, Kanazawa 920-1192, Japan; ^2^Department of Patho-Functional Bioanalysis, Graduate School of Pharmaceutical Sciences, Kyoto University, Kyoto 606-8501, Japan

## Abstract

Nuclear medicine bone imaging has been the optimum diagnosis for the detection of bone disorders because the lesion could be detectable before the appearance of symptomatic and radiographic changes. Over the past three decades, ^99m^Tc-MDP and ^99m^Tc-HMDP have been used as bone scintigraphic agents because of their superior biodistribution characteristics, although they are far from optimal from a chemical and pharmaceutical point of view. Recently, a more logical drug design has been proposed as a concept of bifunctional radiopharmaceuticals in which the carrier molecules (bisphosphonates) and radiometal chelating groups are separated within a molecule, specifically, ^99m^Tc-mononuclear complex-conjugated bisphosphonate. Some of the ^99m^Tc-mononuclear complex-conjugated bisphosphonate compounds showed superior biodistribution in preclinical studies. Moreover, the drug design concept could be applied to ^68^Ga PET bone imaging agents. These studies would provide useful information for the development of radiometal-based imaging and therapeutic agents for bone disorders such as bone metastases.

## 1. Background

The skeleton is one of the most common organs to be affected by metastatic cancer. Carcinomas of the breast, lung, prostate, kidney, and thyroid have a tendency to easily metastasize to bone [1]. Although there has been significant advancement in imaging technologies, such as CT and MR, nuclear medicine bone imaging has been the optimum diagnosis for the detection of bone disorders, such as bone metastases, because of its high sensitivity. Namely, bone-seeking radiopharmaceuticals usually localize in skeletal lesions before the appearance of symptomatic and radiographic changes and the resulting easy evaluation of the entire skeleton [2]. This paper reviews currently available ^99m^Tc radiopharmaceuticals for bone scintigraphy and advances in drug design of radiometal-based bone-targeted compounds.

## 2. ^99m^Tc-Bisphosphonate Complexes

**Figure 1:**
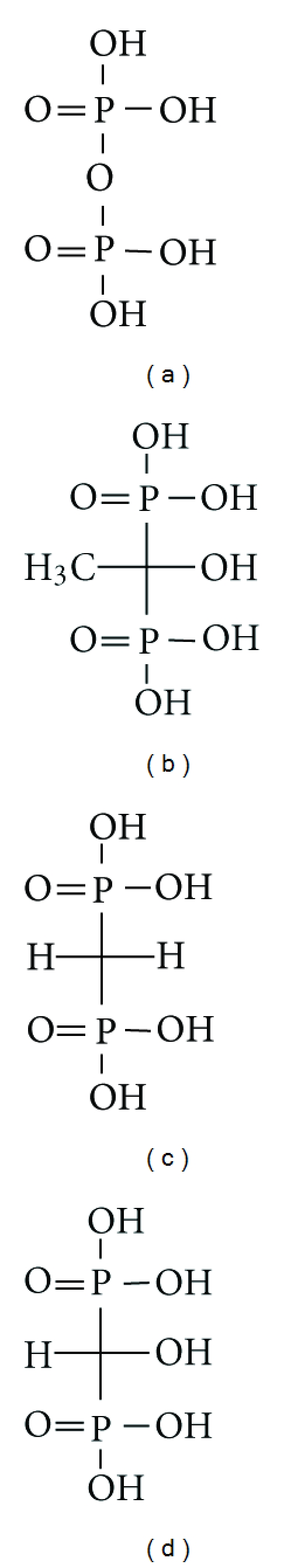
Chemical structures of bisphosphonates analogs (a) pyrophosphate, (b) HEDP, (c) MDP, and (d) HMDP.

Although some radiometals, such as lanthanide and rare earth, localize in bone by themselves, pertechnetate (^99m^TcO_4_^−^) hardly accumulates in bone by itself. Accordingly, a carrier for bone is necessary in order to take bone images with technetium. The first bone-seeking ^99m^Tc compound, a complex of reduced ^99m^Tc and sodium tripolyphosphate, was reported in 1971 [3], followed by a long-chain linear polyphosphate [4] and pyrophosphate [5]. Pyrophosphate (Figure 1(a)) is composed of only two phosphate moieties and is the simplest polyphosphate. ^99m^Tc-pyrophosphate is now seldom used for skeletal imaging because of its high soft tissue background activity but is still employed to determine myocardial infarction. Unfortunately, pyrophosphate and polyphosphate are susceptible to *in vivo* degradation by enzymes such as alkaline phosphatases, resulting in the release of free technetium from the complexes. Subsequently, three groups almost simultaneously reported the ^99m^Tc complex of 1-hydroxyethyliden-1,1-diphosphonate (HEDP, Figure 1(b)) as a new bone imaging agent [6–8]. HEDP is one of the bisphosphonate (diphosphonate) compounds, which are known as compounds with high affinity for bone and inhibitors of bone resorption. Bisphosphonate compounds were synthesized with a P–C–P bonding sequence instead of the P–O–P sequence of pyrophosphate. Although these two chemical structures are similar, the P–C–P bond angles are smaller (117 degrees) than the P–O–P bond angles (128.7 degrees) and the P–C interatomic distance (1.79 Å) is longer than that of P–O (1.63 Å) [9]. Bisphosphonate bonding is very stable chemically and affords greater resistance to *in vivo* phosphatase hydrolysis. As a result, ^99m^Tc-HEDP exhibited more rapid clearance from blood and higher uptake in bone. In clinical study, ^99m^Tc-HEDP showed significantly higher lesion-to-normal bone ratios when compared with either ^99m^Tc-pyrophosphate or ^99m^Tc-polyphosphate [10].

After introduction of ^99m^Tc-methylene diphosphonate (MDP, Figure 1(c)) by Subramanian et al. [11] in 1975 and ^99m^Tc-hydroxymethylene diphosphonate (HMDP, Figure 1(d)) by Bevan et al. [12] in 1980, ^99m^Tc-MDP, and ^99m^Tc-HMDP, which showed superior biodistribution compared to ^99m^Tc-HEDP, have been used as radiopharmaceuticals for bone scintigraphy for over thirty years [13–15]. ^99m^Tc-MDP is postulated to form a bidentate-bidentate bridge with hydroxyapatite while the presence of the hydroxyl group in ^99m^Tc-HMDP would convert the ligand to form a bidentate-tridentate bridge and is expected to enhance the hydroxyapatite affinity of the ^99m^Tc complex [12, 16]. However, the lower bone accumulation of ^99m^Tc-HEDP is not well understood because ^99m^Tc-HEDP should also form bidentate-tridentate binding. Increasing steric hindrance associated with the methyl group at the central carbon atom of HEDP, the difference in solubility, and differences in molecular size as well as in the bisphosphonate polymeric complexes themselves have all been suggested [12, 17].

The accumulation of ^99m^Tc-bisphoshonate complexes in bone must be derived from the coordination of bisphosphonate to calcium in the hydroxyapatite of bone, but the mechanism of high uptake to lesion sites in bone has not been completely elucidated. One factor should be the increased vascularity and regional distribution of blood flow that results from disease. However, it has been shown that regional bone blood flow alone does not account for the increased uptake of radiopharmaceuticals [18]. Other factors are involved in their binding and interaction with bone. It is generally assumed that ^99m^Tc-bisphoshonate complexes accumulate at sites of active bone metabolism, that is to say, at areas of new bone formation or calcification [19, 20]. It has also been reported that the accumulation mechanisms might be both adsorption onto the surface of hydroxyapatite in bone and incorporation into the crystalline structure of hydroxyapatite [21]. Newly formed bone has a much larger surface area than does stable bone. That is, the crystalline structure of hydroxyapatite in newly formed bone is amorphous and has a greater surface area than that in normal bone [22]. An *in vitro* study demonstrated that bisphosphonate compounds have significantly higher adsorption on amorphous calcium phosphate than on crystalline calcium phosphate [17].

Bisphosphonate compounds form multiple complexes with reduced ^99m^Tc. By using high-performance liquid chromatography (HPLC), the relative composition of ^99m^Tc-bisphosphonate complexes in a reaction mixture has been found to vary with pH and with technetium, and with oxygen concentrations [23]. It has been postulated that ^99m^Tc-bisphosphonate complexes would be a mixture of monomers, oxo-bridged dimers, and oligomeric clusters with varying technetium-oxo core configurations, oxidation states, and ligand coordination numbers [24]. These radiolabeled species have different biodistribution properties. It was reported that the smallest, low-charged, mononuclear ^99m^Tc-bisphosphonate complex has the greatest uptake in bone lesions and the highest lesion-to-muscle and lesion-to-normal bone ratios in experiments using each isolated complex by HPLC [25]. Thus, the exact structures and mechanisms of the action of ^99m^Tc-labeled bisphosphonate remain uncertain.

## 3. New Drug Design Concept of ^99m^Tc-Labeled Bisphosphonate (^99m^Tc Complex-Conjugated Bisphosphonate Compounds)

As mentioned above, despite over three decades of clinical use of ^99m^Tc-bisphosphonate complexes, these radiopharmaceuticals are far from optimal from a chemical and pharmaceutical point of view. For example, their structures and compositions remain unknown because they cannot be obtained as a well-defined single-chemical species, but as mixtures of short-chain and long-chain oligomers. The biological behavior of this type of tracer is also affected by the different degrees of ionization and by variations in the relative amount of oligomers after preparation [23].

In addition, in clinical studies, an interval of 2 to 6 hours is required between an injection of ^99m^Tc-labeled bisphosphonates and obtaining bone images [15]. Shortening this interval would lessen the burden to patients in terms of total examination length and radiation dose absorbed. To enable imaging at an earlier time after injection, a radiopharmaceutical with higher affinity for bone might be advantageous. Although the accumulation of bisphosphonate compounds in bone is achieved by binding the phosphonate groups with the Ca^2+^ of hydroxyapatite crystals [26], the phosphonate groups in ^99m^Tc-MDP and ^99m^Tc-HMDP serve as both coordinating ligands and Ca^2+^ binding functional groups [27], which might decrease the inherent accumulation of MDP and HMDP in bone.

**Figure 2:**
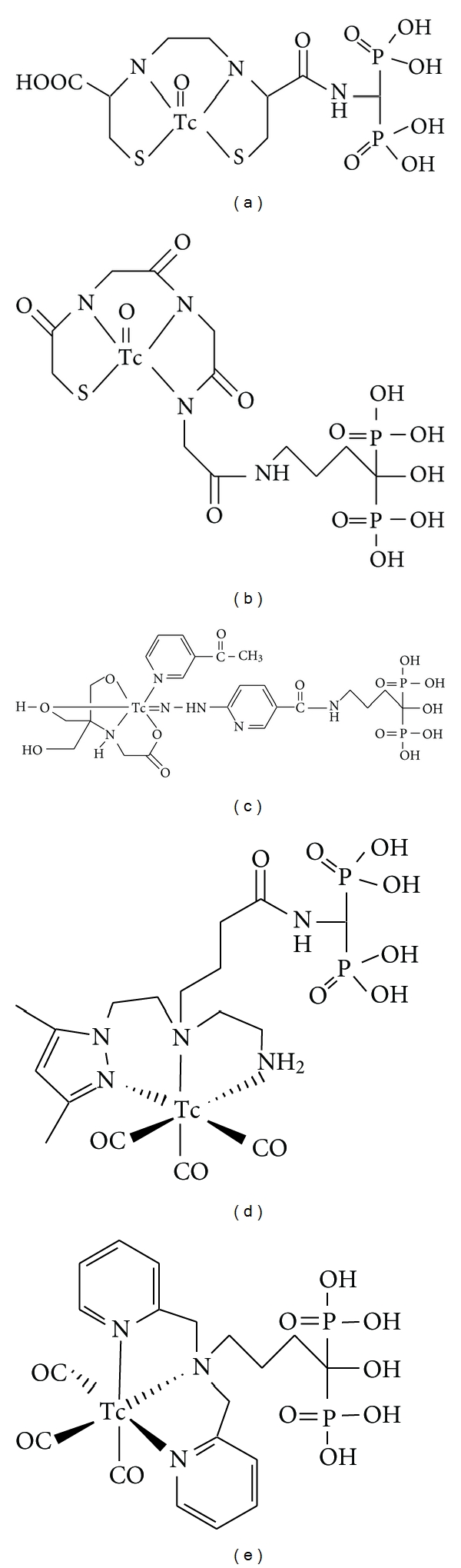
Chemical structures of Tc-complex-conjugated bisphosphonate compounds (a) Tc-ECAMDP, (b) Tc-MAG3-HBP, (c) Tc-HYNIC-HBP, (d) Tc(CO)_3_(*κ* ^3^-pz-BPOH)]^+^, and (e) Tc(CO)_3_-DPA-alendronate.

Recently, to improve the ^99m^Tc-labeled bisphosphonates currently used, a more logical drug design has been proposed based on the concept of bifunctional radiopharmaceuticals in which the carrier molecules (bisphosphonate) and radiometal chelating groups are separated within the molecule so that they can each function independently and effectively. In particular, ^99m^Tc-mononuclear complex-conjugated bisphosphonate compounds have been reported [28–31]. It was hypothesized that the bone affinity of ^99m^Tc labeled bisphosphonate would be enhanced by conjugating a stable mononuclear ^99m^Tc chelating group with a bisphosphonate moiety so that the conjugation does not impair the inherent chemical and biological properties of the bisphosphonate compounds. ^99m^Tc-L,L-ethylene dicysteine (EC), ^99m^Tc-mercaptoacetylglycylglycylglycine (MAG3), ^99m^Tc-6-hydrazinonicotinic acid (HYNIC), ^99m^Tc-tricarbonyl anchored by pyrazolyl- (pz-) containing ligand, and ^99m^Tc-tricarbonyl dipicolylamine (DPA) were selected as ^99m^Tc chelating molecules, and were conjugated with bisphosphonate compounds, (^99m^Tc-ECAMDP, ^99m^Tc-MAG3-HBP, ^99m^Tc-HYNIC-HBP, [^99m^Tc(CO)_3_(*κ* ^3^-pz-BPOH)]^+^, and ^99m^Tc(CO)_3_-DPA-alendronate, resp., Figure 2).

In the drug design of the ^99m^Tc-mononuclear complex-conjugated bisphosphonate compounds, since these ligands contain a bisphosphonate site, there is a possibility that ^99m^Tc coordinates not with the proposed metal coordination moiety, such as EC, MAG3, and HYNIC but with the bisphosphonate moiety. To ascertain whether ^99m^Tc is chelated with only the proposed metal coordination moiety, some experiments were performed. For example, in the case of ^99m^Tc-HYNIC-HBP, ^99m^Tc-HYNIC-HBP was also prepared by the coupling of ^99m^Tc-HYNIC previously complexed with the bisphosphonate site (prelabel method). RP-HPLC analysis revealed the ^99m^Tc-HYNIC-HBP by the prelabel method to be identical to that obtained from the labeling of HYNIC-HBP with ^99m^Tc. These findings exclude the possibility of complexation between technetium and the bisphosphonate structure, and indicate the chelation of ^99m^Tc with the HYNIC moiety in HYNIC-HBP.

In these new compounds, ^99m^Tc-MAG3-HBP, ^99m^Tc-HYNIC-HBP, and ^99m^Tc(CO)_3_-DPA-alendronate were investigated for *in vitro* hydroxyapatite binding as an index of bone affinity. ^99m^Tc-MAG3-HBP and ^99m^Tc-HYNIC-HBP showed a significantly higher rate of binding to hydroxyapatite than did ^99m^Tc-HMDP. ^99m^Tc(CO)_3_-DPA-alendronate showed a higher affinity to hydroxyapatite than did ^99m^Tc-MDP. At the same time, all new ^99m^Tc-mononuclear complex-conjugated bisphosphonate compounds exhibited high bone uptake in *in vivo* animal experiments. ^99m^Tc-EC-AMDP and ^99m^Tc-HYNIC-HBP showed especially superior results; ^99m^Tc-EC-AMDP and ^99m^Tc-HYNIC-HBP showed significantly higher bone-to-blood ratios of radioactivity than did ^99m^Tc-MDP and ^99m^Tc-HMDP.

## 4. Radiogallium-Labeled Compounds as Bone Imaging Agents for PET

^68^Ga is one of the greatest practical and interesting radionuclides for clinical positron emission tomography (PET) because of its radiophysical properties (*T*_1/2_ = 68 min) [32]. ^68^Ga is a generator-produced nuclide and can be eluted at any time on demand. Specifically, it does not require an on-site cyclotron. In principle, the long half-life of the parent nuclide ^68^Ge (*T*_1/2_ = 270.8 days) provides a long life-span generator.

Investigations of ^68^Ga-labeled compounds for bone imaging were previously reported in the 1970s [33, 34]. In these reports, gallium was labeled with tripolyphosphate or ethylenediamine tetramethylene phosphonate (EDTMP) or diethylenetriamine pentamethylene phosphonate (DTPMP). These complexes showed high uptakes in bone. However, since use of the PET camera generally did not spread in the 1970s and the quality of PET cameras was not high, the attention given to ^68^Ga PET imaging agents was not so high.

For the last decade, ^68^Ga as a nuclide has been considered a useful radionuclide for PET imaging. Thus, many ^68^Ga-labeled compounds have been developed. Recently, ^68^Ga-EDTMP was also reevaluated by Mitterhauser et al. [35]. However, they stated that the advantage of ^68^Ga-EDTMP over [^18^F]-fluoride was not apparent and that the future clinical prospect of ^68^Ga-EDTMP remained speculative.

**Figure 3:**
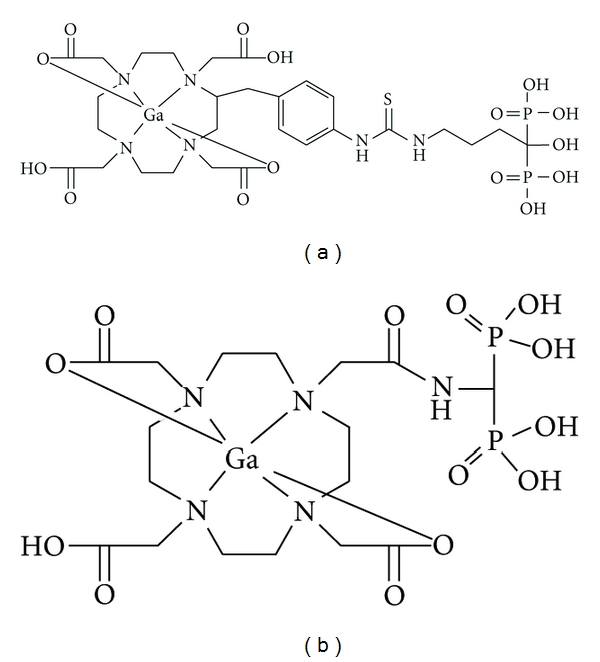
Chemical structures of radiogallium complex-conjugated bisphosphonate compounds (a) Ga-DOTA-Bn-SCN-HBP and (b) Ga-BPAMD.

**Figure 4:**
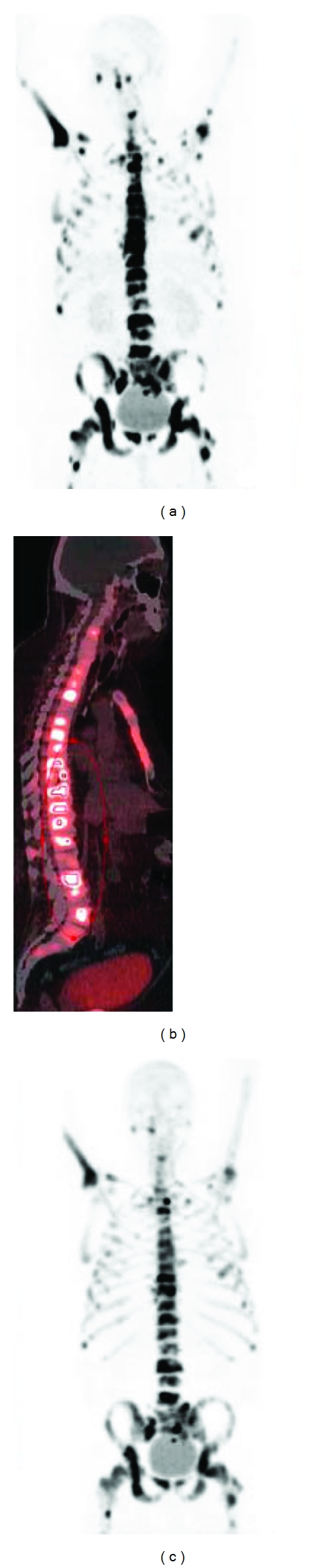
^68^Ga-BPAMD was injected i.v. into a patient with known extensive bone metastases of prostate cancer. ^68^Ga-BPAMD (maximum intensity projection (MIP) 50 min after injection (p.i.), 462 MBq) revealed intense accumulation in multiple osteoblastic lesions in the central skeleton, ribs, and proximal extremities: (a) = coronal PET, (b) = sagittal PET/CT. For comparison, (c) shows ^18^F-fluoride PET (sagittal, MIP 90 min p.i., 270 MBq). With kind permission from Springer Science + Business Media: [36].

The above-mentioned drug concept of stable mononuclear complex-conjugated bisphosphonate could be applicable to not only technetium complex radiopharmaceuticals but also to gallium radiopharmaceuticals. To develop a new PET tracer with radiogallium for imaging bone disorders such as bone metastases, 1,4,7,10-tetraazacyclododecane-1,4,7,10-tetraacetic acid (DOTA) was chosen as a chelating site because it has been well known that Ga forms a stable complex with DOTA. Therefore, Ga-DOTA-conjugated bisphosphonate compounds (^67^Ga-DOTA-Bn-SCN-HBP and ^68^Ga-BPAMD, Figure 3) have been developed [36, 37]. Actually, in biodistribution experiments, ^67^Ga-DOTA-Bn-SCN-HBP rapidly accumulated in bone but was rarely observed in tissues other than bone. In addition, PET/CT imaging of bone metastases with ^68^Ga-BPAMD showed high uptake in osteoblastic metastases of human (Figure 4). The maximal standardized uptake was 77.1 and 62.1 in the 10th thoracic and L2 vertebra versus 39.1 and 39.2 for ^18^F-fluoride PET, respectively. These results suggest that the drug design concept of radiogallium complex-conjugated bisphosphonate could be useful for the development of ^68^Ga PET imaging agents for bone disorders such as bone metastases.

## 5. ^18^F-Fluoride as Bone Imaging Agent for PET

^18^F-fluoride was initially reported by Blau et al. in 1962 [38]. After the development of  ^99m^Tc-labeled bone scintigraphy agents, such as ^99m^Tc-MDP, ^18^F-fluoride was replaced by them because the physical characteristics of  ^99m^Tc were more convenient for imaging with conventional gamma cameras in those days. However, in the last decade, PET and PET/CT have evolved significantly and become widespread. The situation has similarly changed for ^68^Ga-labeled compounds. The changes caused the reemergence of ^18^F-fluoride bone imaging with PET because current PET cameras have higher spatial resolution and greater sensitivity than conventional gamma cameras.

Like ^99m^Tc-MDP as mentioned above, it is known that the distribution of ^18^F-fluoride in bone also reflects both blood flow in bone and osteoblastic activity. Once ^18^F-fluoride reaches the surface of the newly formed hydroxyapatite crystals, fluoride anions are isomorphously exchanged with the hydroxyl group in hydroxyapatite (Ca_10_(PO_4_)_6_(OH)_2_) and fluorapatite (Ca_10_(PO_4_)_6_F_2_) is formed [39]. A previous paper reported that electron probe X-ray fluorescence studies on the topographical distribution of fluoride at the microscopic level in the iliac bone of an osteoporotic patient being treated with fluoride [40]. The results indicate that the distribution of ^18^F-fluoride in newly mineralized bone is similar to that of  ^99m^Tc-MDP.

 There is an important difference between ^18^F-fluoride and ^99m^Tc-MDP in terms of their protein binding rates. ^18^F-fluoride barely binds to serum protein [41] whereas ^99m^Tc-MDP shows significant protein bindings. The difference in protein binding causes a difference in blood clearance between ^18^F-fluoride and ^99m^Tc-MDP. Hence, an interval of 2-3 hours is needed between an injection of  ^99m^Tc-MDP and bone imaging. In contrast, bone imaging can be performed less than 1 hour after an injection of ^18^F-fluoride. Another difference between ^18^F-fluoride and ^99m^Tc-MDP is in terms of uptake to blood cells. ^18^F-fluoride is taken up by red blood cells. The erythrocyte concentration of ^18^F-fluoride is approximately 45–50% of the plasma concentration, namely, approximately 30% of total blood concentration. However, ^18^F-fluoride is freely diffusible from red blood cells to the bone surface, so the uptake of ^18^F-fluoride to red blood cells should not interfere with the accumulation of ^18^F-fluoride in bone [41].

 In clinical use in oncology, some papers reported on a comparison between ^18^F-fluoride PET and planar ^99m^Tc-MDP scintigraphy in the detection of bone metastases [42, 43]. ^18^F-fluoride PET was more sensitive in detecting bone metastases than planar ^99m^Tc-MDP scintigraphy. However, the cause of increased sensitivity—whether it was derived from ^18^F-fluoride itself as a tracer or because of the improved performance of the PET camera—could not be determined.

 Specifically, ^18^F-fluoride PET has two important advantages in diagnosis imaging over planar ^99m^Tc-MDP scintigraphy: superior sensitivity and a shorter interval between injection of a tracer and bone imaging. Thus, the use of ^18^F-fluoride could become common in the future.

## 6. Conclusion

Over the past three decades, ^99m^Tc-MDP and ^99m^Tc-HMDP have been used for detecting bone metastases, although their mechanisms of accumulation remain uncertain. Recent efforts of chelate-conjugated bisphosphonates and their derivatives have provided chemically well-characterized new ^99m^Tc-labeled bone-seeking tracers. Furthermore, the drug design concept could be applied to ^68^Ga PET bone imaging agents. These studies would provide useful information for the development of radiometal-based imaging and therapeutic agents for bone disorders such as bone metastases.
